# P-478. Impact of Active Methicillin-Susceptible Staphylococcus aureus Surveillance and Decolonization in a NICU

**DOI:** 10.1093/ofid/ofaf695.693

**Published:** 2026-01-11

**Authors:** Catherine Foster, Judith R Campbell, Krystal Purnell, Josalyn Curl, Elizabeth Tocco, Lucila Marquez

**Affiliations:** Baylor College of Medicine, Houston, TX; Baylor College of Medicine, Houston, TX; Texas Children's Hospital, Houston, Texas; Texas Children's Hospital, Houston, Texas; Texas Children's Hospital, Houston, Texas; Baylor College of Medicine, Houston, TX

## Abstract

**Background:**

Neonates colonized with *Staphylococcus aureus* are at increased risk of invasive disease. Compared to methicillin-resistant *S. aureus*, data on methicillin-susceptible *S. aureus* (MSSA) colonization and disease among neonates is relatively lacking. While many strategies for *S. aureus* screening and decolonization in neonatal intensive care units (NICUs) exist, an optimal approach and regimen has not been established.

**Figure:**
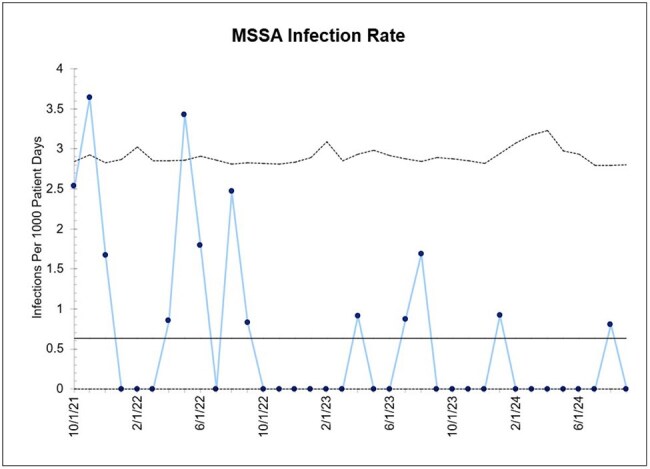

**Methods:**

We performed active surveillance for MSSA colonization during fiscal years 2022-2024 in a 42-bed NICU in Houston, TX. Surveillance swabs from nares, axilla, and groin were tested using polymerase chain reaction with reflex to culture. Infants were screened at admission and weekly. Infants screening positive for MSSA were decolonized with intranasal mupirocin twice daily for five days and, if ≥ 36 weeks gestational age or ≥ 4 weeks chronologic age, bathed with 2% chlorhexidine gluconate (CHG) wipes. The electronic medical record was reviewed for clinical and microbiologic data. Descriptive statistics and Fisher’s exact were used for analysis.

**Results:**

The overall MSSA colonization rate was 9.7% (281 of 2902 screened infants) with a median time to colonization of 14 days (range 0-77). Infants colonized with MSSA had significantly lower birthweights and gestational ages compared to non-MSSA colonized infants (*P*< 0.001 for both). Two hundred and fourteen (76%) of MSSA-colonized infants completed the decolonization protocol with mupirocin and the most frequent reason for partial or no mupirocin decolonization was patient discharge. Only 133 (47%) of MSSA-colonized infants were eligible to receive CHG. Twenty-four of 281 (8.5%) colonized infants developed a MSSA infection, including 18 infants (75%) with bacteremia. Infection rate over the study period is shown in Figure 1. Of the infants (n=28) that received 5 days of CHG decolonization, none developed MSSA infection, while twenty of 238 (8.4%) who did not receive CHG developed MSSA infection (*P*=0.14).

**Conclusion:**

Among neonates, colonization with MSSA is relatively common. In our study, adherence to decolonization with mupirocin was much higher than with CHG. The observed infection rate did not decrease post our screening and decolonization intervention.

**Disclosures:**

All Authors: No reported disclosures

